# NSD-SSD: A Novel Real-Time Ship Detector Based on Convolutional Neural Network in Surveillance Video

**DOI:** 10.1155/2021/7018035

**Published:** 2021-09-08

**Authors:** Jiuwu Sun, Zhijing Xu, Shanshan Liang

**Affiliations:** ^1^College of Information Engineering, Shanghai Maritime University, Shanghai 201306, China; ^2^School of Physics and Electronics, Shandong Normal University, Jinan 250358, China

## Abstract

With the rapid development of the marine industry, intelligent ship detection plays a very important role in the marine traffic safety and the port management. Current detection methods mainly focus on synthetic aperture radar (SAR) images, which is of great significance to the field of ship detection. However, these methods sometimes cannot meet the real-time requirement. To solve the problems, a novel ship detection network based on SSD (Single Shot Detector), named NSD-SSD, is proposed in this paper. Nowadays, the surveillance system is widely used in the indoor and outdoor environment, and its combination with deep learning greatly promotes the development of intelligent object detection and recognition. The NSD-SSD uses visual images captured by surveillance cameras to achieve real-time detection and further improves detection performance. First, dilated convolution and multiscale feature fusion are combined to improve the small objects' performance and detection accuracy. Second, an improved prediction module is introduced to enhance deeper feature extraction ability of the model, and the mean Average Precision (mAP) and recall are significant improved. Finally, the prior boxes are reconstructed by using the *K*-means clustering algorithm, the Intersection-over-Union (IoU) is higher, and the visual effect is better. The experimental results based on ship images show that the mAP and recall can reach 89.3% and 93.6%, respectively, which outperforms the representative model (Faster R-CNN, SSD, and YOLOv3). Moreover, our model's FPS is 45, which can meet real-time detection acquirement well. Hence, the proposed method has the better overall performance and achieves higher detection efficiency and better robustness.

## 1. Introduction

With the rapid development of the shipping industry, there are more frequent human activities on the ocean in recent years. Therefore, robust ship detection is strongly needed to meet the demand. Currently, ship detection is used in port transportation management, sea area monitoring over illegal activities, and ship abnormal behavior detection for navigation safety. Modern radar target tracking equipment and ship automatic identification systems are mainly based on positioning, and thus, ship detection needs substantial improvements. In response to these problems, many researchers have used traditional machine learning methods to explore this field in search of better results. For example, they used features of ships combined with classifiers [1, 2]. Although these methods achieve good results, they require manual extraction of features and a classifier with good performance, which needs further validation in terms of efficiency and accuracy. Fortunately, the development of deep learning has enabled object detection to be widely used in many scenarios, such as surveillance security and autonomous driving. In 2019, Jiao et al. [3] provided a comprehensive analysis of the current state and future trends of deep learning-based object detection. Convolutional Neural Networks (CNN) can effectively learn the corresponding features from massive samples, which avoids the complicated feature extraction process and achieves higher accuracy. In 1998, Lecun et al. [4] proposed LeNet-5 and achieved success in the recognition of handwritten characters. Since then, the performance of CNNs has been improved with the appearance of deeper and more complex CNNs such as AlexNet [5], VGGNet [6], GoogLeNet [7], ResNet [8], and DenseNet [9]. In 2020, Abdollahi et al. [10] used a generative adversarial network (GAN) architecture to extract building footprints from high-resolution aerial images. However, the algorithms of regular CNNs combined with feature pyramid networks (FPN) have become a new focus in the field of object detection. The object detection algorithms currently mainly include two technical routes: two-stage detection and one-stage detection. The two-stage detection is divided into two steps. First obtain the region proposals, and then, these region proposals are classified and regressed to get the final detection results. Two-stage detectors mainly include R-CNN [11], SPP-Net [12], Fast R-CNN [13], Faster R-CNN [14], and Mask R-CNN [15]. For one-stage detection, it treats the object detection problem as a regression problem. A unified CNN completes the object classification and location, which is an end-to-end target detection solution. One-stage detectors mainly include OverFeat [16], SSD [17], and YOLO [18–21]. Many scholars proposed improved YOLOv3 and SSD for object detection and obtained outstanding detection performance [22, 23]. The two-stage detection algorithm such as Faster R-CNN has high accuracy, but its region proposal network (RPN) is time-consuming and therefore reduces the detection efficiency. On the contrary, although the YOLO series has a great advantage in terms of detection speed, they cannot achieve high accuracy.

The SSD is used as a one-stage detector and introduces a multiscale feature layer for object detection, which has faster detection speed but accuracy needs to be improved. In this paper, the SSD is applied to ship detection and several improvements are used to improve the overall performance of the network.

(1) To address the problem of poor performance of small target detection, we apply a dilated convolution on the low-level feature layer to expand the receptive field so that the low-level feature layer can also contain more feature information. At the same time, we perform multiscale fusion on the original feature layers after up-sampling so that the network can make full use of the contextual information. (2) We introduce a residual structure in the prediction module of the network to enable the network to extract deeper dimensional feature information for better classification and regression. (3) We use the *K*-means clustering algorithm to reconstruct the prior bounding box so as to obtain a more suitable scale and aspect ratio, which can improve both the visual effect and the efficiency of ship detection. Finally, we propose a new SSD-based network, called NSD-SSD, which is significantly better than the original SSD. Compared with SSD and other detection networks, the proposed network provides a good trade-off between real-time detection and accuracy.

The rest of this paper is organized as follows. In Section 2, we introduce the related work of the ship detection. In Section 3, we give detailed program of our proposed approach.  Section 4 outlines the experimental results and comparisons against other state-of-the-art methods. Finally, conclusions are made in Section 5.

## 2. Related Work

This paper categorizes the previous work of ship object detection to traditional methods and deep learning methods.

The traditional detection methods include two types. (1) Ship-radiated noise-based methods: Kang et al. [24] proposed a multiple classifier fusion algorithm based on many-person decision theory to identify ship radiated noise, with accuracy rate of over 96%. Zhao et al. [25] proposed a decision tree support vector machine (SVM) classification method based on the ship-radiated noise multidimension feature vector for the measured radiated noise of three kinds of ship targets. Luo and Wang [26] used the time-frequency range characteristics of ship noise to distinguish ship's stern, ship's mid-aft, and ship's middle part to complete the positioning and identification of ship targets. Peng et al. [27] proposed a ship-radiated noise model based on the winger's higher-order spectrum for feature extraction. (2) Ship structure and shape characteristics-based methods: Zhu et al. [28] proposed a novel hierarchical method of ship detection from spaceborne optical image based on shape and texture features, and this method can effectively distinguish ships from nonships on the optical image dataset. Liu et al. [29] used segmentation and shape analysis to detect inshore ships and proved their method was effective and robust under various situations. Shi et al. [30] proposed an approach involving a predetection stage and an accurate detection stage to detect ships in a coarse-to-fine manner in high-resolution optical images. Wang et al. [31] proposed a detection method based on DoG (Difference of Gaussian) preprocessing and shape features to detect ship targets in remote sensing images.

Most of the traditional methods use manually extracted features, which will lead to low efficiency and high time consumption. At the same time, even if a classifier with good performance is used to classify these features, the accuracy cannot meet the actual demand. Therefore, the recognition rate of these methods in complex environmental background and multivessel classification is not ideal.

The deep learning detection methods: with the boom development of deep learning, many ship object detection methods based on deep CNN have been proposed. Zou et al. [32] proposed an improved SSD algorithm based on MobilenetV2 [33] and finally achieved better detection results in three types of ship images. Zhao et al. [34] proposed a new network architecture based on the Faster R-CNN by using squeeze and excitation for ship detection in SAR images. Shao et al. [35] proposed a saliency-aware CNN framework and coastline segmentation method to improve the accuracy and robustness of ship detection under complex seashore surveillance conditions. Nie et al. [36] proposed an improved Mask R-CNN model, which can accurately detect and segment ships from remote sensing images at the pixel level. Guo et al. [37] proposed a novel SSD network structure to improve the semantic information by deconvoluting high-level features into a low-level feature and then fusing it with original low-level features, and the model performed well on both the PASCAL VOC and railway datasets. Huang et al. [38] proposed a new network by referring to the feature extraction layer of YOLOv2 and feature pyramid network of YOLOv3, and the new network model can detect seven types of ships. Zhao et al. [39] proposed the Attention Receptive Pyramid Network (ARPN), which detected multiscale ships in SAR images. Li et al. [40] proposed a new method, combining the Saliency Estimation Algorithms (SEAs) and the Deep CNN (DCNN) object detection to ensure the extraction of large-scale ships. In 2021, Zhao et al. [41] proposed a feature pyramid enhancement strategy (FPES) and a cascade detection mechanism to improve SSD, and the improved model can be applied to vehicle detection quickly and efficiently.

In short, although the existing ship target detection methods have made major breakthroughs, they still have certain limitations. Firstly, the low-level feature map contains less semantic information but can accurately present the location of the target. In contrast, high-level feature maps contain rich semantic information but cannot accurately display the location of objects. In addition, the previous methods cannot extract the features of small objects well. In this paper, we use a multiscale feature fusion algorithm, which considers the ability of the entire network to combine the context information and improve small target detection performance. In addition, we have also improved the prediction module and the settings of prior boxes. Finally, we test the improved model on the ship dataset.

## 3. Materials and Methods

### 3.1. Single-Shot Multibox Detector

**Figure 1:**
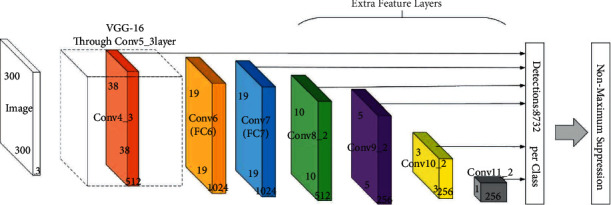
SSD network structure diagram.

Figure 1 shows the SSD network structure diagram with a backbone network VGG-16. VGG-16 has stable network structure and good feature extraction capabilities. The SSD network converts FC6 and FC7 in VGG-16 into convolutional layers, removes all Dropout layers and FC8 layers, and adds four additional convolutional layers: Conv6, Conv7, Conv8, and Conv9. The feature pyramid structure is to detect objects of different sizes. In the process of detection, a large number of prior boxes are usually generated, and these prior boxes have multiple predefines scales and ratios. Finally, it is required to apply a Nonmaximum Suppression (NMS) process to obtain the final test results. The biggest advantage of the SSD network is that classification and regression are carried out at the same time, which improves the detection speed compared with other models such as Faster R-CNN.

### 3.2. Our Proposed Network

**Figure 2:**
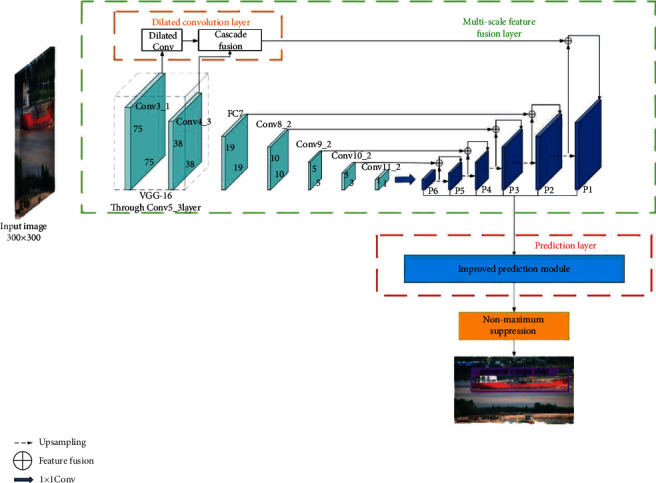
The architecture of the proposed novel ship detection SSD (NSD-SSD).

The overall architecture of the Novel Ship Detection SSD (NSD-SSD) is shown in Figure 2. From the figure, the architecture mainly is formed by three parts, a dilated convolution layer, a multiscale feature fusion layer, and a prediction layer. In addition, the prior boxes are reconstructed within this network. Ship images are sent to the NSD-SSD network for a series of operations, and finally, the specific location and type of ship can be obtained.

**Figure 3:**
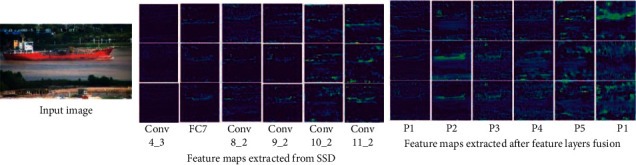
Feature maps of ship images extracted by the network.

To understand the features extracted by the network more clearly, a visualization of the feature maps is given in Figure 3. In the figure, from left to right, the input image, the feature maps extracted by SSD, and the feature maps extracted after feature layer fusion are shown. From the figure, we can see that the feature maps extracted by the original SSD network lack rich semantic information. For example, the main characteristics of the low-level feature layer Conv4_3 are small perceptual field and too poor ability to extract target features. However, after the dilated convolution and features fusion, the feature information of the target is greatly enriched. Similarly, all other scale layers also extract a large amount of meaningful contextual information after feature fusion, which greatly improves the accuracy of object detection.

#### 3.2.1. Dilated Convolution Layer

Traditional SSD network mainly uses low-level feature layer Conv4_3 to detect small objects. However, due to insufficient feature extraction in the Conv4_3 layer, the detection effect of small objects is not ideal. To address this issue, we use dilated convolution to map high-dimensional features to low-dimensional input. In this paper, we choose the lower-level feature layer Conv3_1 for dilated convolution and merge it with Conv4_3 for feature fusion. In this way, the range of the receptive field can be enlarged without loss of image detail information and obtains more global information.

Dilated convolution is to inject dilation on map of the standard convolution to increase the receptive field. The dilated convolution has another hyperparameter called the dilation rate, which refers to the number of intervals of the convolution kernel. Assuming that the original convolution kernel is *f* and the dilation rate is *α*, the new convolution kernel size *n* after dilated convolution is

(1) $$\begin{array}{c} n=\alpha \times \left(f-1\right)+1. \end{array}$$

The receptive field size *r* after dilated convolution is

(2) $$\begin{array}{c} r=\left[2^{\left(\alpha /2\right)+2}-1\right]\times \left[2^{\left(\alpha /2\right)+2}-1\right]. \end{array}$$

Suppose that there is a dilated convolution with *f*=3 and *α*=1, which is equivalent to a standard convolution. Its receptive field is 3 × 3. When *f*=3 and *α*=2, according to equations (1) and (2), its new convolution kernel is 5 × 5, and the receptive field size is expanded to 7 × 7 without losing detailed information.

**Figure 4:**
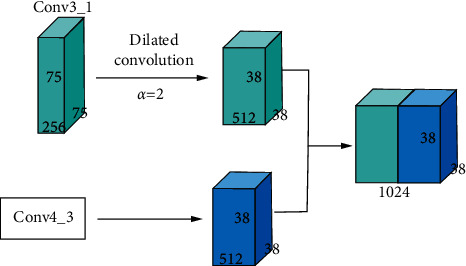
Fusion process of the Conv3_1 layer with the Conv4_3 layer after dilated convolution.

In this paper, we choose the Conv3_1 layer for dilated convolution. The original kernel is 3 × 3, stride is 2, pad is 2, and dilation rate is 2. From equation (1), the new convolution kernel is 5 × 5. The original feature map of Conv3_1 layer is 75 × 75 × 256. After performing dilated convolution, it obtains a feature map size which is 38 × 38 × 512. From equation (2), the receptive filed is 7 × 7. The Conv3_1 layer undergoes feature map fusion with the Conv4_3 layer after dilated convolution. There are two main ways of feature map fusion: additive fusion and cascade fusion. Because the cascade fusion has a small amount of calculation and high accuracy, in this paper, we choose cascade fusion method.  Figure 4 shows the process of feature map fusion.

**Figure 5:**
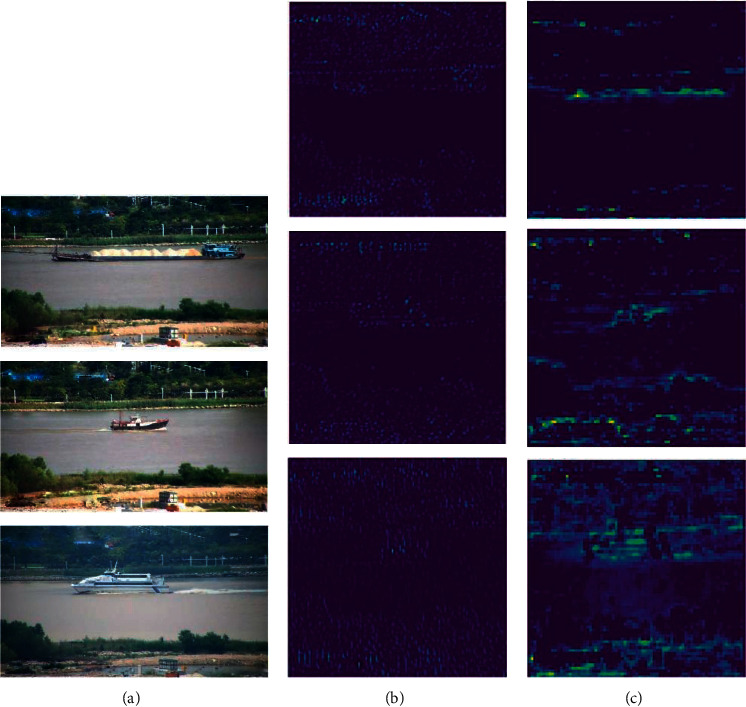
Comparison of the feature maps in the original SSD and after the dilated convolution.

To better explain how the dilated convolution improves the performance of the network with the addition of feature maps, Figure 5 shows the feature maps of the image before and after the dilated convolution.

In the figure, (a) is the original image, (b) are the feature maps of Conv4_3 in the SSD network, and (c) are the feature maps with dilated convolution and feature fusion. The original features of the Conv4_3 activation area and perceptual field are small and cannot detect the ship targets at the corresponding scales well. The original features of the Conv4_3 activation area and perceptual field are small and cannot detect the ship targets at the corresponding scales well. On the contrary, the dilated convolution and feature fusion are able to more richly extract the texture and detail features on the low-level feature maps, and the contours and shapes are more clearly distinguished.

#### 3.2.2. Multiscale Feature Fusion Layer

The original SSD network uses the Feature Pyramid Network (FPN) to detect different feature layers so that it can adapt to different object sizes. Although this detection method provides the possibility of multiscale object detection, it does not consider the combination of shallow features and deep features. In this study, on the basis of the original SSD network, we introduce a multiscale feature fusion mechanism. This method can synthesize shallow high-resolution features and deep semantic features to make joint decisions. The green dotted box in Figure 2 shows the specific fusion connections of different feature layers. The left half of the figure is the original SSD network feature layer, and the right half is the fused feature layer. The specific implementation process of this feature fusion method will be described in detail below. First, perform 1 × 1 convolution of Conv11_2 to obtain P6, then perform up-sampling of P6, and finally perform 1 × 1 convolution of Conv10_2 with the feature layer obtained by up-sampling P6 to obtain P5. The purpose of up-sampling here is to obtain the feature map of the size required for fusion. After the same fusion process, the fused feature layers are successively P4, P3, P2, and P1. In this way, the combination of shallow features and deep features is considered comprehensively, and it is possible to improve the detection accuracy. P1 is formed by fusion of dilated convolutional layer and P2 up-sampling. The parameters of the prediction layer are shown in Table 1.

#### 3.2.3. Improved Prediction Module

The SSD network uses a set of convolution filters at each effective feature layer to obtain prediction results. For each effective feature layer with a size of *h* × *w* with *d*channels, use a 3 × 3 convolution operation on each route to obtain the score of each category and the change of each prior bounding box.

**Figure 6:**
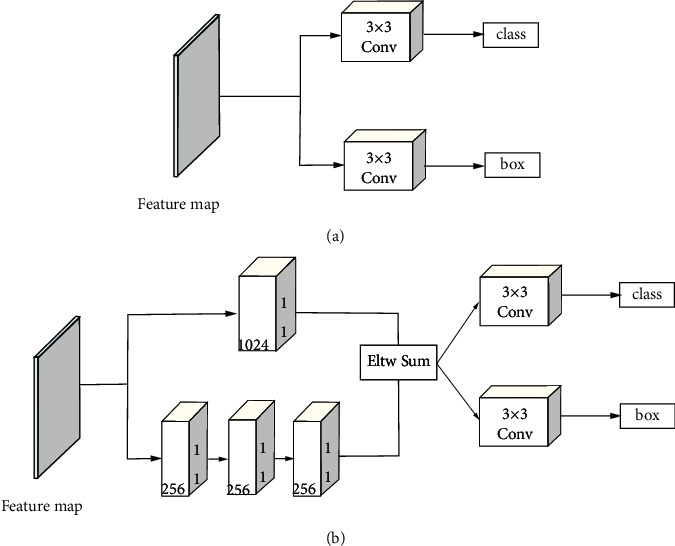
The prediction process of the feature layer. (a) The original SSD predictor: obtain the score of each category and the change of the prior box after two convolution routes. (b) The improved predictor: add the residual structure on the basis of (a) to obtain the prediction result.

MS-CNN [42] points out that improving the subnetwork of each task can improve the accuracy. DSSD [43] follows this principle and proposes an improved prediction module, and experimental results show that this method can improve detection accuracy. Therefore, we transplant the idea of DSSD into our network model to better improve the detection performance. The prediction layer corresponds to the red box in Figure 2. That is, on the basis of SSD, the original structure is changed to a residual module. The residual prediction block allows the use of 1 × 1 convolution to predict the score of each category and the changes of prior boxes. The structure of the original predictor and the improved predictor are shown in Figure 6. In this way, deeper dimensional features can be extracted for classification and regression.

#### 3.2.4. Reconstruction of Regional Prior Box

The performance of deep learning object detection algorithms largely depends on the quality of feature learning driven by training data. In the SSD object detection task, the training data is the regional prior box. The SSD network has selected a total of 6 effective feature layers as the prediction layer, the sizes of which are (38, 38), (19, 19), (10, 10), (5, 5), (3, 3), and (1, 1), but the number of a prior bounding boxes set on each feature map is different. The prior bounding box has two hyperparameters: scale and aspect ratio. The scale of a prior bounding box in each prediction layer is

(3) $$\begin{array}{c} S_{k}=S_{\min}+\frac{S_{\max}-S_{\min}}{m-1}\left(k-1\right),\;k\in \left[1,m\right]. \end{array}$$

Among them, *m* refers to the number of feature maps (*m*=6 in the SSD algorithm), *s*_*k*_ represents the ratio of the prior box size of the *k*th feature map to the picture, *S*_min_ represents the minimum value of the ratio, and the value is 0.2, and *S*_max_ indicates the maximum value of the ratio, and the value is 0.9. The aspect ratio of the prior bounding box is generally set to *a*_*r*_={1,2,3, 1/2, 1/3}. The width and height of the prior bounding box are as follows:

(4) $$\begin{array}{c} \left\{\begin{array}{c} w_{k}^{a}=S_{k}\sqrt{a_{r}},\\ \overset{a}{\underset{k}{h}}=\frac{S_{k}}{\sqrt{a_{r}}}. \end{array}\right) \end{array}$$

By default, each feature map will have a prior bounding box with *a*_*r*_=1 and a scale of *S*_*k*_. In addition, the prior bounding box with a scale of $S_{k}^{\prime }=\sqrt{S_{k}S_{k+1}}$ will be added. In this way, each feature map has two square prior bounding boxes with an aspect ratio of 1 but different sizes. The maximum side length of the square prior bounding box is $S_{k}^{\prime }=\sqrt{S_{k}S_{k+1}}$, and the minimum side length is *S*_*k*_.  Table 2 lists the min-size and max-size of the prior bounding boxes used in this paper.

**Figure 7:**
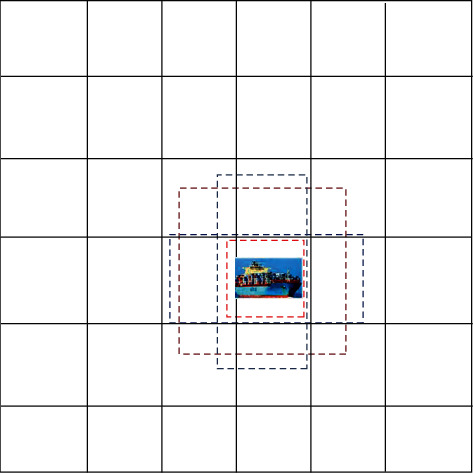
Schematic diagram of the prior bounding box. At this time, the aspect ratio *a*_*r*_={1,2}, and there are 4 prior bounding boxes.

As shown in Figure 7, 4 prior bounding boxes are generated, two squares (red dashed line) and two rectangles (blue dashed line). At this time, the aspect ratio *a*_*r*_={1,2}. Among them, *S*_*k*_*∗*300 is the side length of the small square and $\sqrt{S_{k}S_{k+1}}*300$ is the side length of the large square. 300 is the size of the input image in the SSD algorithm. The width and height of the corresponding two rectangles are

(5) $$\begin{array}{c} \left\{\begin{array}{c} \sqrt{a_{r}}*S_{k}*300,\;\frac{1}{\sqrt{a_{r}}}*S_{k}*300,\\ \sqrt{\frac{1}{a_{r}}}*S_{k}*300,\;\frac{1}{\sqrt{1/a_{r}}}*S_{k}*300. \end{array}\right) \end{array}$$

When 6 prior bounding boxes are to be generated, the aspect ratio *a*_*r*_={1,2,3}. The center point of each prior box is (*i*+0.5/|*f*_*k*_|, *j*+0.5/|*f*_*k*_|), *i* and *j* ∈ [0, |*f*_*k*_|], and *f*_*k*_ is the size length of the feature map. In this paper, *f*_*k*_={38,19,10,5,3,1}.  Table 3 shows the detailed parameters of the prior bounding boxes of the SSD algorithm.

In the SSD algorithm, the scale and aspect ratio of the prior boxes in the network cannot be obtained through learning, but manually set. Since each feature map in the network uses different prior bounding boxes in scale and shape, the debugging process is very dependent on experience. In this paper, we use the *K*-means algorithm to predict the scale and proportion of the prior bounding box to improve the detection efficiency of the network. The standard *K*-means clustering algorithm uses Euclidean distance to measure distance. But if Euclidean distance is used here, the larger boxes will produce more errors than the small boxes. Therefore, we use other distance measurement methods, and the specific equation is as follows:

(6) $$\begin{array}{c} d\left(\text{box},\text{centroid}\right)=1-\text{IOU}\left(\text{box},\text{centroid}\right)\\ =1-\text{IOU}\left[\left(x_{j},y_{j},w_{j},h_{j}\right),\left(x_{j},y_{j},W_{i},H_{i}\right)\right]. \end{array}$$

IoU is the intersection ratio between the regional prior bounding boxes and the ground truth boxes, and we expect a larger IoU. The purpose of clustering is that the prior bounding boxes and the adjacent ground truth have a large IoU value. Equation (6) just ensures that the smaller the distance, the larger the IoU value.

In this paper, we will traverse different types of labeled boxes in the dataset and cluster different types of boxes. Some specific parameters in equation (6) are as follows: (*x*_*j*_, *y*_*j*_, *w*_*j*_, *h*_*j*_), *j* ∈ {1,2,…, *k*}, is the coordinates of the label boxes. (*x*_*j*_, *y*_*j*_) is the center point of the box, (*w*_*j*_, *h*_*j*_) is the width and height of the boxes, and *N* is the number of all label boxes. Given *k* cluster center points (*W*_*i*_, *H*_*i*_), *i* ∈ {1,2,…, *k*}, where *W*_*i*_ and *H*_*i*_ are the width and height of the prior bounding box. Calculate the distance between each label box and each cluster center, and the center of each label box coincides with the cluster center during calculation. In this way, the label box is assigned to the nearest cluster center. After all the label boxes are allocated, the cluster centers are recalculated for each cluster. The equation is as follows:

(7) $$\begin{array}{c} W_{i}^{\prime }=\frac{1}{N_{i}}\sum w_{i},\\ H_{i}^{\prime }=\frac{1}{N_{i}}\sum h_{i}, \end{array}$$

where *N*_*i*_ is the number of label boxes in the *i*th cluster, that is, find the average of all label boxes in the cluster. Repeat the above steps until the cluster center changes very little.

**Figure 8:**
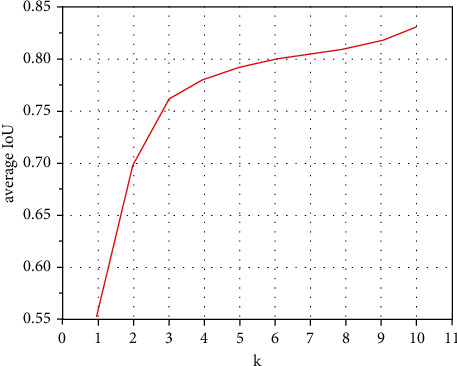
The clustering map of the prior bounding box.

In this paper, we set the number of cluster center *k* = {0, 1, 2, 3, 4, 5, 6, 7, 8, 9, 10} to conduct experiments and use the average IoU to measure the results of the experiment, so as to complete the reconstruction of the prior box. It can be seen from Figure 8 that when *k* ≤ 6, the average IoU increases greatly, and when *k* > 6, it basically tends to be flat. By combining the calculation amount of the entire algorithm for comprehensive consideration, we choose *k*=6. At this time, the aspect ratio of the prior bounding box is predicted to be [0.35, 0.89, 1.18, 1.69, 1.89, 2.86].  Table 4 shows the specific parameters of the prior bounding box setting in the NSD-SSD algorithm. Through the method of prior bounding box reconstruction, the error of the algorithm is reduced with improved accuracy and efficiency.

### 3.3. Loss Function

When training the detection network, we need to measure the error between the candidate boxes and the truth value boxes and minimize this error. At this time, for each candidate box, the offset of the center point of the candidate box relative to the center of the truth box and the confidence of the candidate box needs to be calculated. In the training phase, there are generally two samples, called positive samples and negative samples. Here, we consider the matching value of the candidate box and the truth box to be greater than the threshold as positive samples, denoted by *d*^1^, and other candidate boxes that do not satisfy minimum matching value are considered negative samples, denoted by *d*^2^. In order to ensure the balance of the sample, the ratio of positive and negative samples is required to be at most 3 : 1.

The loss function of the NSD-SSD algorithm is basically similar to that of the SSD. In this study, the total loss function includes the classification loss and the localization loss:

(8) $$\begin{array}{c} L\left(x,c,l,g\right)=\frac{1}{N}\left(L_{\text{cls}}\left(x,c\right)+\alpha L_{\text{loc}}\left(x,l,g\right)\right), \end{array}$$

where *N* is the number of the positive samples. If *N*=0, we set the loss to 0. *c* is confidence, *l* is the predicted box, and *g* is the ground truth box. *α* is the balance coefficient between classification loss and localization loss, and its value usually is 1.

The localization loss is smooth *L*1 loss, *x*_*ij*_^*p*^ is an indicator, and *x*_*ij*_^*p*^={0,1}. When *x*_*ij*_^*p*^=1, it means that the *i*th candidate box matches *j*th ground truth box of ship category *p*:

(9) $$\begin{array}{c} L_{\text{loc}}\left(x,l,g\right)=\overset{N}{\underset{i\in d^{1}}{\sum }}\underset{m\in \left\{cx,xy,w,h\right\}}{\sum }x_{ij}^{k}\text{smooth}L1\left(l_{i}^{m}-{\hat{g}}_{j}^{m}\right), \end{array}$$

where

(10) $$\begin{array}{c} \text{smooth}L1\left(x\right)=\left\{\begin{array}{cc} 0.5x^{2} & \left|x\right|<1,\\ \left|x\right|-0.5 & \text{otherwise}. \end{array}\right) \end{array}$$

**Figure 9:**
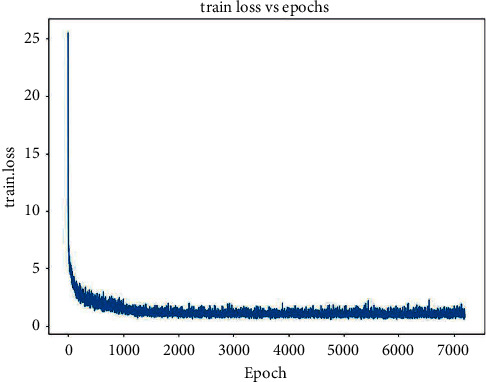
The loss function curve of the NSD-SSD.

The classification loss is the Softmax loss. When classifying, the confidence level belonging to the ship category *p* is expressed by *c*^*p*^, and the confidence level belonging to the background is expressed as *c*^0^:

(11) $$\begin{array}{c} L_{\text{cls}}\left(x,c\right)=-\sum_{i\in d^{1}}^{N}x_{ij}^{p}\log\left({\hat{c}}_{i}^{p}\right)-\sum_{i\in d^{2}}\log\left({\hat{c}}_{i}^{0}\right), \end{array}$$

where ${\hat{c}}_{i}^{p}=\text{exp}\left(c_{i}^{p}\right)/\sum_{p}\exp\left(c_{i}^{p}\right)$. In the first half of equation (11), the predicted frame *i* and the real frame *j* match with respect to the ship category *p*. The higher the predicted probability of *p*, the smaller the loss. In the second half of the equation, there is no ship in the predicted box. That is, the higher the predicted probability of the background, the smaller the loss. In this study, we use Stochastic Gradient Descent to optimize the loss function to find the optimal solution. The final loss function curve of NSD-SSD is shown in Figure 9. Note that due to the result of deep learning in this model, the loss function in the early stage will fluctuate, but it will eventually become stable.

## 4. Experimental Results

To prove the effectiveness of our proposed method, we designed experiments and quantitatively evaluated the proposed method on the public ship dataset. Subjective and objective results will be presented in this section, and the results will also be analyzed.

### 4.1. Dataset

**Figure 10:**
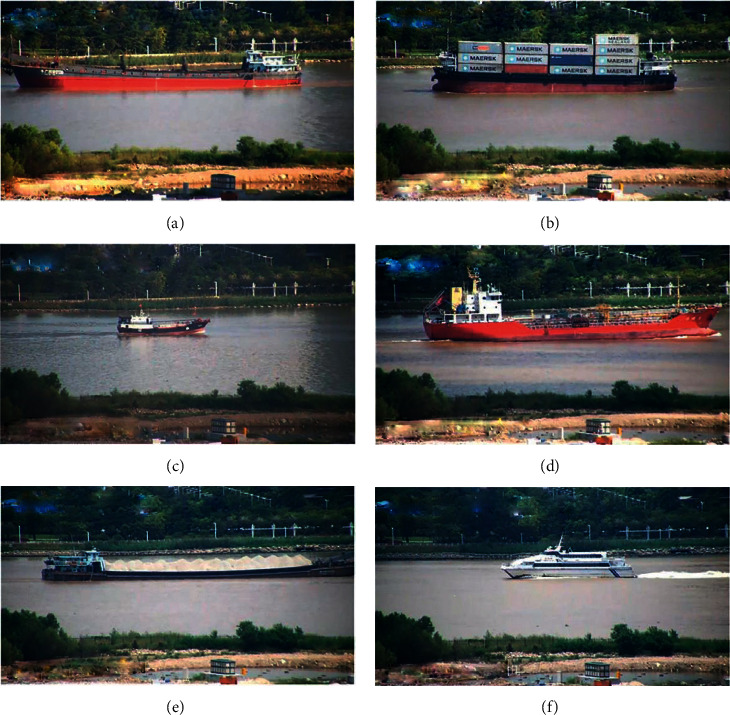
The ship category in our used dataset. (a) Bulk cargo carrier. (b) Container ship. (c) Fishing boat. (d) General cargo ship. (e) Ore carrier. (f) Passenger ship.

In this paper, we use a public dataset called SeaShips [44] for ship detection. This dataset consists of 6 common ship categories and 7000 images in total, including ore carrier, bulk cargo carrier, general cargo ship, container ship, fishing boat, and passenger ship. All of the images are video clips taken by surveillance camera, covering all possible imaging changes, with different proportions, hull parts, background, and occlusion. All images are marked with ship category labels and bounding boxes. The example images of each ship category are shown in Figure 10. In order to better train and evaluate the model, we divided the dataset into a training set, a validation set, and a testing set. The 3500 images were randomly selected as the training set, 1750 images as the validation set, and the rest as the testing set. In particular, the validation set was useful to avoid overfitting for better model selection.

### 4.2. Test Settings

All the models in our experiment are run on a 64-bit Ubuntu operating system using a 2.9 GHz Intel Core-i5 with 15.6 GB of RAM and NVIDIA GTX 1080Ti GPU with 11 GB of video RAM. The deep learning framework that we use is Pytorch which runs on GPU.

Our proposed network structure is modified from SSD, and the NSD-SSD and SSD use the same hyperparameters for training. The batch size we used is 32, and the num_workers is 4. The initial learning rate is set to 0.001. Momentum is 0.9, and weight decay is 0.0002.

### 4.3. Evaluation Index

Since this article studies the task of ship object detection, several mature indicators are needed to evaluate the detection model. These indicators will be described in detail below.

(1) Intersection-over-Union (IoU): IoU is a standard for measuring the accuracy of detecting the position of corresponding objects in a specific dataset. In other words, this standard is used to measure the correlation between real and predicted. The higher the correlation, the greater the value. The equation is as follows:
  (12) $$\begin{array}{c} \text{IoU}=\frac{G_{t}\cap D_{r}}{G_{t}\cup D_{r}}. \end{array}$$
In equation (12), *G*_*t*_ is the ground-truth bounding box, *D*_*r*_ is the predicted bounding box, *G*_*t*_∩*D*_*r*_ is the intersection of *G*_*t*_ and *D*_*r*_, and *G*_*t*_ ∪ *D*_*r*_ is the union of *G*_*t*_ and *D*_*r*_. The range of IoU is 0-1; in this paper, we set the threshold to 0.5. Once the IoU calculation result is greater than 0.5, it is marked as a positive sample; otherwise, it is also a negative sample.
(2) Average precision: After the IoU threshold is given, there will be two indicators called precision and recall. The precision refers to the number of ground truth ships in all predictions. The recall refers to the number of ground truth ships predicted in all ground truth ships. So, precision and recall are as follows:
  (13) $$\begin{array}{c} \text{precision}=\frac{\text{TP}}{\text{TP}+\text{FP}},\\ \text{recall}=\frac{\text{TP}}{\text{TP}+\text{FN}}. \end{array}$$
According to precision and recall, a precision-recall curve can be drawn, referred to as the PR curve. AP is the area enclosed by this curve, and the specific equation is as follows:
  (14) $$\begin{array}{c} \text{AP}=\int_{0}^{1}P\left(R\right)\text{d}R, \end{array}$$
(3) mean Average Precision (mAP): mAP shows the average values of AP_*i*_ of each class *i*:
  (15) $$\begin{array}{c} \text{mAP}=\frac{\sum_{i=1}^{n}{\text{AP}}_{i}}{n}. \end{array}$$
Here, *n* represents the number of classes of ships that need to be detected.
(4) *Frames Per Second (FPS)*: the FPS is used to judge the detection speed of different models, and the larger the FPS value, the faster the speed.

### 4.4. Results and Analysis

Our model is based on the SSD network with the backbone of VGG16. To test the detection performance of NSD-SSD, the comparative experiments are implemented using several popular baseline methods: Faster R-CNN, SSD series, and YOLO series. The backbone networks of these models are pretrained on ImageNet. To achieve a fair comparison, we train and test the four models using the same dataset. At the same time, to ensure the consistency of training, we set the hyperparameters and the number of training echoes of these three baseline models to be the same as the NSD-SSD.

**Figure 11:**
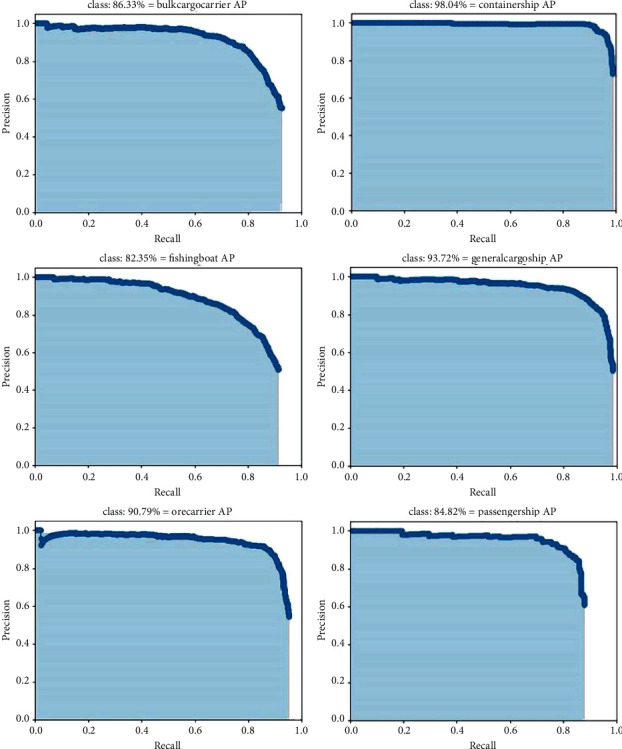
Precision-recall curves of our proposed method (NSD-SSD) on six categories of ships.

According to our detection method (NSD-SSD), the AP performance of the six categories of ship is shown in Figure 11. The IoU threshold is set to 0.5 in the experiment.

We record the accuracy of the four models based on the evaluation indicators, as shown in Table 5. The detection performance of Faster R-CNN is significantly better than YOLO series and SSD series. On average, Faster R-CNN's mAP is 22.5% and 17.8% higher than SSD series and 12.5% and 8.2% higher than YOLO series, respectively. Although our proposed model (NSD-SSD) has a little gap with Faster R-CNN in mAP, our approach significantly improves the performance of SSD. Moreover, it performs better than Faster R-CNN on general cargo ship.

Our proposed method is based on SSD (VGG16). The detection effect of the original SSD network is indeed not good, and the accuracy is extremely average. But compared with SSD, the mAP of each category of ship in our model has a good improvement and the NSD-SSD's mAP is 20.2% higher than original SSD. Among six categories of ships, the container ships have the best detection results. Because they mainly transport containers, and these cargoes have very distinct shape characteristics that are different from other ships. The ore carriers also achieve excellent detection results. Because they usually transport ore, they have the special features like container ships. In addition, since the general cargo ships are very large in the images, their results are also extremely good. On the contrary, the performance of fishing boats is the worst among these six categories of ships. The main season is that fishing boats are too small, occupying a few pixels in the 1920 × 1080 image. Detectors are generally not good at detecting small objects. After layers of convolution, the feature information of small objects will become blurred, and even the SSD model is worse.

**Figure 12:**
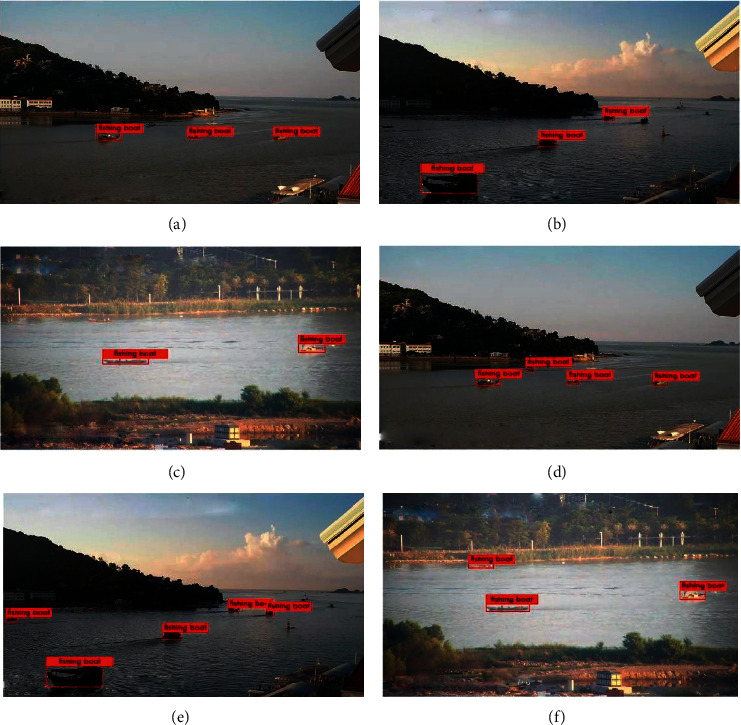
Some fishing boats' detection results. (a–c) The original SSD. (d–f) Our proposed method.

We perform structural improvements on the basis of SSD and add detailed detection techniques, which makes it possible for us to better detect small targets and improve the overall accuracy. For fishing boats, we have increased from 60.4% to 82.4%, which already exceeds the mAP of the YOLOv3 model. As shown in examples in Figure 12, our proposed method greatly improves the detection effect of fishing boats against SSD. For the passenger ships, our method has increased by nearly 10%. For the general cargo ships, our method makes their performance become better and has a significant improvement over Faster R-CNN.

In terms of detection speed, FPS of 24 is called the standard for real-time detection in object detection. As can be seen from Table 6, the detection speed of YOLOv3 is much better than other detection model, and the FPS reaches 79. Unfortunately, its detection effect is not good. The detection speed of SSD series can be ranked second, and the FPS can reach 75 and 68.0, respectively, but detection performance is worse. Since the Faster R-CNN is a two-stage detector, the detection process is more complicated, which results in its FPS of only 7 and cannot meet real-time detection. Our proposed model adds many parameters and calculations on the basis of SSD, thereby reducing the speed. The FPS given by our method is 45, which not only guarantees the real-time detection requirements but also improves the detection accuracy. In addition, we also give the parameters of IoU and recall for different models, and our method is better than other methods.

**Figure 13:**
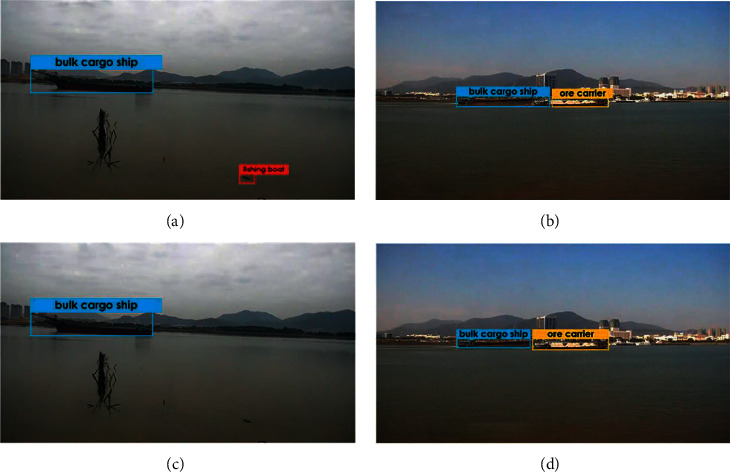
Ship detection results. (a) The Faster R-CNN. (b) YOLOv3. (c, d) Our proposed model.

In Figure 13 we show the detection examples of our model against Faster R-CNN and YOLOv3, and our proposed method has a better visual effect. Specifically, when the two ships are very close together, the bounding box of YOLOv3 is much larger or smaller than ship, but our method can mark a more accurate box. Furthermore, the Faster R-CNN sometimes detects the background as a ship, but our proposed method can avoid the false detection.

We compare the proposed method with [35], and they propose a detection method that combines YOLOv2 and saliency detection and achieve good results. The comparison results are shown in Table 7. From the table, our method is slightly better than the comparison method on mAP. Among the six categories of ships, container ships and fishing boats can achieve better results. Specifically, these two categories of ships' AP is 7.7% and 4.1% higher than the comparison method, respectively. For passenger ships, our method is 3.8% lower than Shao's method because the color characteristic of passenger ships is very salient, the performance of their proposed saliency detection is particularly good, and the accuracy is higher. In addition, the IoU of our method is higher and the detection visual effect is better, but the FPS of Shao's model is 4 higher than the FPS of our model.

To verify the effectiveness of our proposed various modules, we conduct the ablation experiment for comparison, and the original SSD is the baseline network. Moreover, our proposed three modules are considered as setting items, and the experimental results are shown in Table 8.

As can be seen from the table that the detection accuracy of SSD is 10.7% higher than that of the backbone network VGG16, indicating that SSD is a better detection network. When introducing the feature fusion in SSD, the mAP has increased from 69.1% to 83.2%. Because our algorithm considers the combination of shallow features and deep features and makes full use of contextual information. When adding the remaining two parts of modules, the mAP has increased by 6.1%. The above results prove that our proposed method can effectively improve the accuracy of ship detection.

**Figure 14:**
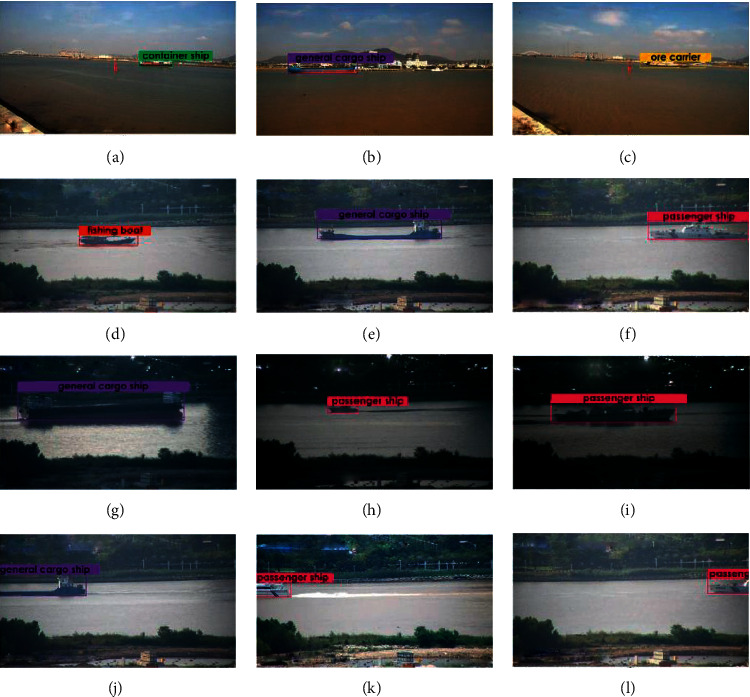
Visualization of ship detection with the proposed method under different conditions. (a–c) Sunny. (d–f) Rainy. (g–i) Night. (j–l) Incomplete ship.

Furthermore, we validate our proposed method under practical extreme conditions, as shown in Figure 14, and under different weather conditions, such as sunny, rainy, and night. On the contrary, the ships in the images are incomplete. However, our method still achieves excellent detection performance, and the marked bounding boxes and the classifications are reasonable and accurate, respectively.

## 5. Conclusion

In this paper, based on real-time ship detection task as our basic goal as well as the characterization of the ship dataset, a novel ships' detector in visual images captured by the monitoring sensor, named NSD-SSD, is proposed. The NSD-SSD is mainly based on multiscale feature fusion (MFF), predicted module (PM), and reconstruction of prior boxes (RPB). Regarding the problem of small objects detection, the dilated convolution is used to expand the receptive field of low-level feature layers, and the network can fully use the contextual information by the MFF. For the problem of setting prior boxes manually, we propose RPB by using the *K*-means clustering algorithm to improve the detection efficiency. In addition, the PM is introduced to extract deeper features. We train our model on the ship dataset and compare it with other conventional methods. The experimental results prove that our proposed method is able to acquire higher accuracy and recall, and it can meet the requirement of real-time detection. Moreover, the NSD-SSD can also guarantee high-quality detection performance in the relatively extreme environment. We also noticed that the method could be improved for ship detection in complex backgrounds. We will address this issue in our future work.

## Figures and Tables

**Table 1:** Parameters of the prediction layer.

Prediction layer	Kernel size	Padding	Kernel numbers	Strides	Feature map
P1	3 × 3	1	1024	1	38 × 38
P2	3 × 3	1	1024	1	19 × 19
P3	3 × 3	1	512	1	10 × 10
P4	3 × 3	1	256	1	5 × 5
P5	3 × 3	1	256	1	3 × 3
P6	3 × 3	1	256	1	1 × 1

**Table 2:** Size of prior bounding boxes for different feature layers.

Feature layer	Min-size	Max-size
Conv4_3	30	60
FC7	60	111
Conv8_2	111	162
Conv9_2	162	213
Conv10_2	213	264
Conv11_2	264	315

**Table 3:** The specific parameters of prior bounding boxes in the SSD algorithm.

Feature map	Size	Numbers	*a*_*r*_
Conv4_3	38 × 38	4	1, 2
FC7	19 × 19	6	1, 2, 3
Conv8_2	10 × 10	6	1, 2, 3
Conv9_2	5 × 5	6	1, 2, 3
Conv10_2	3 × 3	4	1, 2
Conv11_2	1 × 1	4	1, 2

**Table 4:** The specific parameters of prior bounding boxes in the NSD-SSD algorithm.

Feature map	Size	Numbers	*a*_*r*_
Conv4_3	38 × 38	6	1, 2, 3
FC7	19 × 19	6	1, 2, 3
Conv8_2	10 × 10	6	1, 2, 3
Conv9_2	5 × 5	6	1, 2, 3
Conv10_2	3 × 3	6	1, 2, 3
Conv11_2	1 × 1	6	1, 2, 3

**Table 5:** Detection accuracy of different detection models.

Model	mAP	Bulk cargo ship	Container ship	Fishing boat	General cargo ship	Ore carrier	Passenger ship
Faster R-CNN	**0.916**	**0.893**	**0.986**	**0.908**	0.927	**0.914**	**0.868**
SSD (VGG16)	0.691	0.661	0.801	0.604	0.703	0.620	0.755
SSD (Mobilev2)	0.738	0.703	0.876	0.635	0.742	0.686	0.783
YOLOv3	0.791	0.681	0.959	0.690	0.893	0.734	0.786
YOLOv4	0.834	0.849	0.929	0.732	0.851	0.778	0.862
NSD-SSD	0.893	0.863	0.980	0.824	**0.937**	0.908	0.848

**Table 6:** The detection results of other indicators for different detectors.

Model	IoU	Recall	FPS
Faster R-CNN	0.603	0.865	7
YOLOv3	0.616	0.834	**79**
SSD (VGG16)	0.781	0.700	75
SSD (Mobilev2)	0.745	0.787	68
YOLOv4	0.716	0.854	56
Ours	**0.808**	**0.936**	45

**Table 7:** Detection results of different detection models.

Model	IoU	mAP	Bulk cargo ship	Container ship	Fishing boat	General cargo ship	Ore carrier	Passenger ship	FPS
Ours	**0.8082**	**0.893**	0.863	**0.980**	**0.824**	**0.937**	**0.908**	0.848	45
Shao's	0.7453	0.874	**0.876**	0.903	0.783	0.917	0.881	**0.886**	**49**

**Table 8:** The results of the ablation experiment.

VGG16	SSD	Feature fusion	Improved predicted module	Prior boxes reconstruction	mAP
✓					0.584
	✓				0.691
	✓	✓			0.832
	✓	✓	✓	✓	**0.893**

## Data Availability

SeaShip: http://www.lmars.whu.edu.cn/prof_web/shaozhenfeng/datasets/SeaShips(7000).zip (accessed on 2 November 2020).
